# Frequency and Underlying Causes of Alloimmunization Against Red Blood Cell Antigens in Patients Referred to the Blood Bank of the Tertiary Referral Hospital of Tehran from 2018 to 2020

**DOI:** 10.34172/aim.2023.75

**Published:** 2023-09-01

**Authors:** Samaneh Salarvand, Samira Moeini Nasab, Alireza Abdollahi, Zohreh Nozarian, Elham Nazar

**Affiliations:** ^1^Department of Pathology, Imam Khomeini Complex Hospital (IKHC), Tehran University of Medical Sciences, Tehran, Iran; ^2^Department of Pathology, Farabi Hospital, Tehran University of Medical Sciences, Tehran, Iran; ^3^Department of Pathology, Sina Hospital, Tehran University of Medical Sciences, Tehran, Iran

**Keywords:** Alloimmunization, Alloantibody, Blood group, Red blood cell

## Abstract

**Background:**

Alloimmunization against blood group antigens is an important non-infectious complication of blood transfusion, and early detection of these alloantibodies by antibody screening before transfusion is crucial. Identifying which underlying factors will affect the occurrence of alloimmunization will be necessary to manage this event as accurately as possible. We aimed to assess the prevalence rate and main determinants of RBC alloimmunization among patients referred to a large referral blood bank in Iran.

**Methods:**

This retrospective cross-sectional study was conducted on all patients referred to a blood bank at Imam Khomeini Hospital between October 2018 and September 2020. Information was collected by referring to the archives of the hospital information system as well as the documents recorded at the blood bank ward and reviewed by two pathologists and completed documents.

**Results:**

In total, 39270 cases were cross-matched. Accordingly, the frequency of alloimmunization cases was equal to 220 cases, which indicated a prevalence of 0.56%. The most common alloantibodies were anti-K (43.2%, 95% CI: 36.8‒49.5), anti-E (34%, 95% CI: 27.7‒40.5), and anti-C (16.3%, 95% CI: 11.4‒21.4). Among patients with positive alloimmunization, the most common blood groups were blood group B (34.6%), followed by blood group A (34.1%). Most of these patients were Rh-positive (77.3%). In patients with positive alloimmunization, the frequency of hemoglobinopathy was estimated to be 37.7%. Frequent blood transfusions were found in 42.2%, a history of malignancy in 17.3%, graft history in 11.3%, and a history of pregnancy in 35.0%.

**Conclusion:**

Alloimmunization was more prevalent and more predictable among patients with hemoglobinopathies and those receiving recurrent transfusions. Therefore, a history of repeated blood transfusions should be regarded as a risk factor contributing to alloimmunization.

## Introduction

 Most blood group antigens are proteins. Each year in the United States, nearly one in 70 people who receive a transfusion develops an immune response against erythrocyte antigens (alloantigen).^1^

 Except for anti-A and anti-B, which are naturally present in the blood, other alloantibodies against red cell antigens are called unexpected antibodies.^2^ Alloantibodies react only with allogeneic (non-self) red blood cells. Immunization against blood group antigens may ensue pregnancy, blood transfusion, or transplantation.^3^

 Alloimmunization is associated with numerous problems from delays in providing matched blood products to delayed hemolytic reactions after blood transfusion.^4^ Also, alloimmunization can complicate blood transfusion management. In addition, alloimmunization shortens the lifespan of red blood cells, further raising the need for blood products by patients. By identifying the nature of the target antigen subjected to alloimmunization and subsequently transfusing compatible blood products, it is possible to prevent the premature destruction of red blood cells, reduce demands for blood, and avoid the complications associated with recurrent blood transfusions.^5^ The incidence of alloimmunization in frequent blood receivers has been reported at up to 60%.^6,7^

 Numerous studies have estimated that the prevalence of alloimmunization against red blood cells per unit of blood transfusion reaches 1% to 1.6% in the general population and 5% to 21% in patients with recurrent blood transfusions (thalassemia, leukemia, etc).^8^ In patients with hemoglobinopathies (such as beta-thalassemia receiving regular transfusions), the incidence of alloimmunization against red cells antigens, especially against the antigens of the Rh blood group system, is particularly high.^7^

 Alloimmunization-related complications are causes of transfusion-related morbidity and mortality. Both donor- and recipient-related factors, as well as tissue-related parameters play key roles in the incidence of alloimmunization against erythrocytes. A variety of factors, including blood group compatibility and the ability of the recipient to present antigens to the immune system can influence the risk of alloimmunization.^9,10^

 In this study, we aimed to determine alloantibodies against red blood cells antigens in different blood group systems and to present solutions for better identification of these alloantibodies. This can help reduce the immunological ramifications of alloimmunization in people who need blood transfusions.

## Materials and Methods

 The study population included all the patients referred to the blood bank of Imam Khomeini Hospital of Tehran (which can be generalized to Iran’s whole population as this is a national referral center) from October 2018 to September 2020. In this retrospective basic-applied study, the sera obtained from the patients referred to the blood bank of Imam Khomeini Hospital were evaluated for the presence of clinically important alloantibodies. The patients were recruited by the census sampling method. The data (age, gender, medical records, and the type of alloantibodies) were collected by reviewing patients’ medical records available in the hospital’s blood bank. Patients who did not have enough or suitable samples to perform the tests or the patients without available clinical information were excluded from the study. The antibody screening test was performed following the standard protocol using IMMUCOR screening reagent cells (USA).

 In order to provide patients with appropriate blood products, routine ABO and Rh blood grouping tests, as well as antibody screening and cross-matching were performed. Antibodies and albumin were purchased from Immunodiagnostic Co. (Germany). The tests were performed according to the standard protocol. All used antisera and antibody screening vials were tested for quality control assessment and the results were acceptable. The negative results in the anti-human globulin phase were tested using the check cell and had an acceptable result.

###  Statistical Analysis

 The data were analyzed using SPSS 23 applying a 95% confidence interval and using the Chi-square test. The results were expressed using means ± standard deviations (mean ± SD) for quantitative variables and percentages for categorical qualitative variables.

## Results

 Totally, 77 171 cross-matches were performed for 39 270 patient who needed blood transfusion. Out of these, 44,621 blood bags were finally transfused to patients, retrieving a crossmatch to transfusion ratio (C/T) of 1.7. Of the 39 270 patients who underwent antibody screening, 58% (95% CI: 52.3‒65.5) were female, and 42% (95% CI: 35.5‒48.7) were male. Overall, 220 patients showed alloimmunization, indicating a prevalence of 0.56%.

 Among 220 patients with alloimmunization, 102 (46.3%) were female, and 118 (53.7%) were male. The mean age of the patients was 40.37 ± 11.42 years, ranging from 12 to 87 years. In terms of the distribution of major blood groups among patients with alloimmunization, the frequencies of the B, A, O, and AB groups were 43.6% (95% CI: 37.3‒50), 34.1% (95% CI: 22.7‒40.5), 13.6% (95% CI: 9.1‒18.2), and 8.6% (95% CI: 5‒12.3), respectively. Regarding the Rh blood group, the frequencies of D + and D- phenotypes were 77.3% (95% CI: 76.8‒87.3) and 22.7% (95% CI: 12.7‒23.2), respectively. Among patients with alloimmunization, 83 (37.7%) suffered from hemoglobinopathies; 93 (42.2%) had frequent blood transfusions; 38 (17.3%) reported a history of malignancies, and 24 (11.3%) had a history of transplantation. Also, 77 (35%) cases had prior pregnancies.

**Figure 1:**
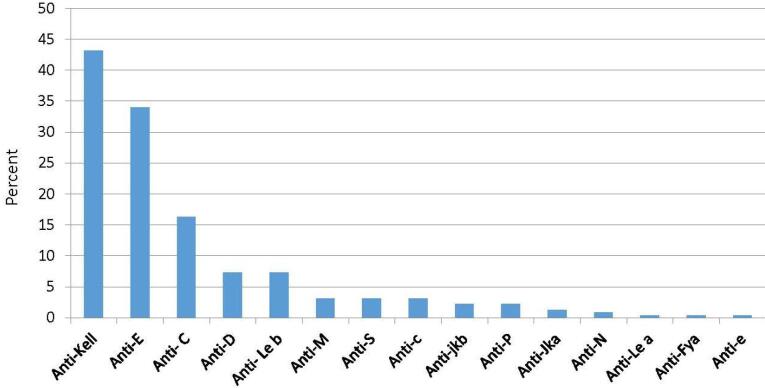
Relative Frequencies of Alloantibodies in Immunized Patients

 Among 220 individuals who had a positive antibody screening test (i.e. alloimmunized), antibodies against the K, E, C, D, Le(b + ), M, c, S, Jkb, P1, Jka, N, Le(a + ), Fya, and e antigens were detected in 95 (43.2% with 95% CI: 36.8‒49.5), 75 (34% with 95% CI: 27.7‒40.5), 36 (16.3% with 95% CI: 11.4‒21.4), 16 (7.3% with 95% CI: 5.9-13.6), 16 (7.3% with 95% CI: 5.9‒13.6), 7 (3.1% with 95% CI:0.9-5.5), 7 (3.1% with 95% CI:0.9‒5.5), 7 (3.1% with 95% CI:0.9‒5.5), 5 (2.2% with 95% CI: 0.5‒4.5), 5 (2.2% with 95% CI:0.5‒4.5), 3 (1.3% with 95% CI: 1.4‒6.4), 2 (0.9%), 1 (0.4%), 1 (0.4%), and 1 (0.4%) cases, respectively (Figure 1).

**Figure 2:**
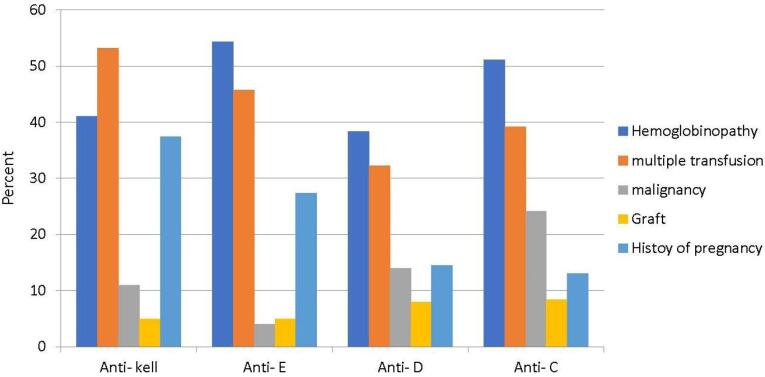
Features and Factors Related to the Development of Alloantibodies

 The distribution of the factors associated with alloimmunization for the most common alloantibodies was as follows (Figure 2):

###  Anti-Kell Antibodies

 Recurrent blood transfusions (53.2%), hemoglobinopathies (41.1%), history of malignancy (11%), history of receiving a graft (5%), and prior pregnancy (37.4%) were the most common factors associated with the occurrence of anti-Kell antibodies.

###  Anti-E Antibodies

 Hemoglobinopathies (54.3%), recurrent blood transfusions (45.7%), history of malignancy (4%), history of receiving a graft (4%), and prior pregnancy (27.4%) were the most common factors associated with the occurrence of anti-E antibodies

###  Anti-D Antibodies

 Among these individuals, 38.4% had hemoglobinopathies, 32.2% had a history of regular blood transfusions, 14% had a history of malignancy, 8% had a history of receiving grafts, and finally, 14.5% had prior pregnancies.

###  Anti-C Antibodies

 Among these patients, 51.1% had hemoglobinopathies, 39.2% had a history of regular blood transfusions, 24.2% had a history of malignancy, 8.4% had a history of receiving grafts, and 13% had a history of pregnancy.

 The results showed that alloimmunized people did not possess necessarily one type of antibody, rather some patients revealed two or more alloantibodies (Table 1). A significant portion of these patients shared risk factors such as frequent blood transfusions for treating hemoglobinopathies, as well as pregnancy and other causes (i.e., multiple risk factors in one person). Nevertheless, as mentioned above, the most common risk factor among alloimmunized individuals in this study was regular blood transfusions (42.2%) for treating hemoglobinopathies (*P* value = 0.001).

**Table 1:** Number of Alloantibodies in Patients with Alloimmunization

**Number of Alloantibodies**	**Number of Patients**	**%**
1	163	74.1
2	45	20.5
3	11	5
4	1	0.5

 Out of 220 individuals detected with alloimmunization in our study, 26% had two or more alloantibodies. Among 83 patients with hemoglobinopathies receiving blood products regularly, the rate of multiple alloantibodies was found to be 14.5%.

 The distribution of main blood groups (ABO) in patients with the most commonly identified alloantibodies was as follows:

*Anti-Kell (95 out of 220, 43.2%):* The frequencies of ABO blood groups were: A (51.9%), B (24.1%), AB (10.3%), and O (13.7%). Regarding the Rh blood group system, the “D” antigen was positive in 82%, while 18% were “D”-negative (*P* value = 0.43).

*Anti-E (75 out of 220, 34.1%):* The frequencies of ABO blood groups was: A (29.4%), B (23.5%), AB (11.7%), and O (35.4%). Regarding the Rh blood group system, the “D” antigen was positive in 91.1%, while 8.9% were “D”-negative (*P* value = 0.26).

*Anti-C (36 out of 220, 16.4%):* The frequencies of ABO blood groups were 20%, 26.6%, 13.3%, and 40.1% for the A, B, AB, and O groups, respectively. Regarding the Rh blood group system, the “D” antigen was positive in 53.3%, while 46.7% were “D”-negative (*P* value = 0.62).

*Anti-D (16 out of 220, 7.3%):* The frequencies of ABO blood groups were 31.3%, 56.2%, and 12.5%, for the A, B, and O groups, respectively. All patients in this group were “D”-negative (*P* value = 0.01).

**Figure 3:**
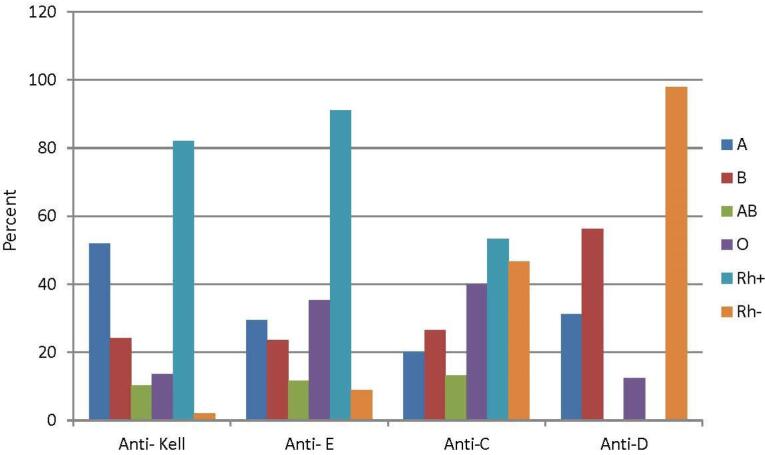
Frequencies of ABO and Rh Blood Groups in Patients with Common Alloantibodies

 As is obvious, there was no significant relationship between the distribution of ABO blood groups and the type of the alloantibodies produced (Figure 3).

## Discussion

 Alloimmunization against blood group antigens is among the most common non-infectious complications of blood transfusion, and the pre-transfusion detection of these alloantibodies by antibody screening is crucial to prevent transfusion reactions. Because of this, in addition to blood grouping, screening tests for unexpected alloantibodies and cross-matching are routinely performed in most medical centers. Alloantibodies against the main and other blood group antigens have variable frequencies, which also influences decisions on choosing and preparing blood products under certain conditions. Therefore, it is necessary to delineate the underlying factors affecting the occurrence of alloimmunization to precisely manage blood transfusion.

 The blood bank of Imam Khomeini hospital of Tehran is one of the largest referral centers for cross-matching blood products. In the present study, we aimed to ascertain the prevalence of alloimmunization against red blood cell antigens and to determine the underlying factors associated with this phenomenon. For this purpose, a total of 39270 patients referred over two years were assessed, of whom 220 cases developed alloimmunization, indicating a prevalence of 0.56%. The prevalence of alloimmunization was similar in men and women, showing no gender deviation in this regard. The peak age spectrum of alloimmunization encompassed the fourth and fifth decades of life. Regarding other underlying features, alloimmunization was mainly detected in patients with either A or B blood group and Rh positivity. Moreover, about one-third of alloimmunized patients suffered from hemoglobinopathies, and about 40% had a history of regular blood transfusions. A narrower ratio of the patients revealed a history of malignancies or transplantation. According to the results of this descriptive study, it seems that blood group, hemoglobinopathies, or recurrent transfusions play a major role in the development of alloimmunization.

 The prevalence of alloimmunization observed in this study was similar to that reported in most studies in Iran and the world. In a study by Ghasemi et al in Iran, the prevalence of alloantibodies between multiply transfused patients of thalassemia major was 10.9 % with the most common types of anti-Kell and Rh.^11^ In a study in Minnesota, Winters et al reported that the prevalence of alloimmunization was less than 1%.^12^ In agreement with our study, Gharehbaghian et al also investigated the prevalence and characteristics of alloantibodies in Tehran, reporting a prevalence of 0.97%, with the most common clinically significant antibodies being anti-K, anti-E, and anti-C.^13^ In a study by Ghorbani Aliabadi et al on the patients needing blood transfusion in Shiraz, 0.7% of male participants and 0.9% of female participants had alloantibodies.^14^ In a 2005 study by Ameen et al on 179 045 blood samples in Kuwait, the prevalence of alloimmunization was reported as 0.49%, and the most prevalent alloantibodies were anti-D, anti-E, and anti-K, respectively.^15^ In a study by Schonewille et al in New Zealand on 564 patients with hematologic malignancies and disorders, 9% of the patients revealed alloimmunization, and the most common alloantibodies were anti-E and anti-C.^16^ Also, in studies conducted in Greece, Italy and the United States, the prevalence of alloimmunization was reported to be 5% to 10% in beta-thalassemia patients requiring regular blood transfusions.^17-19^ Overall, it can be said that the prevalence of alloimmunization varies in different societies depending on demographic factors. As well, hemoglobinopathies and frequent transfusions independently increase the risk of alloimmunization, as confirmed in many studies. In the present study, patients with hemoglobinopathies were under regular blood transfusion protocols, and most of them had the blood group “A” and Rh “D” positivity. Besides, the most frequent alloantibodies observed in this population were against the K (KEL1) (41.3%), E (30.1%), C (22.2%), D (15%), S (2.2%), c (7.7%), and P (2.2%) antigens, respectively. This was consistent with the reports of most previous retrospective studies, including that conducted by Ghorbani Aliabadi et al in Shiraz.^14^

 Considering the relatively higher frequencies of anti-K, anti-E, and anti-C alloantibodies, it is better to give priority to providing blood products that do not have their corresponding antigens. Nevertheless, the antibody screening test is not routinely performed for all patients in some centers, which can lead to some alloimmunized patients remaining unidentified. This is particularly important for patients who are at risk of alloimmunization, such as those receiving recurrent blood transfusions. Occasionally, a positive antibody screening test may occur in a patient who needs blood transfusion promptly and with no delays. Considering the prevalence of the alloantibodies observed in this study, it may be possible to crossmatch packed cells that do not express the antigens corresponding to these common antibodies, allowing for the emergency transfusion of compatible blood units. Our results suggest a link between the blood group “B” and the development of alloimmunization, which requires further investigations in future studies.

## Conclusion

 The overall prevalence of alloimmunization against red blood cell antigens in Iran was 0.56%. The most common alloantibodies were anti-K, anti-E, and anti-C. Alloimmunization was more prevalent and more predictable among patients with hemoglobinopathies and those receiving recurrent transfusions. Therefore, a history of repeated blood transfusions should be regarded as a risk factor contributing to alloimmunization. Identifying the factors predisposing to the development of alloantibodies will help manage blood product preparation and ensure the transfusion of safe and compatible blood products to at-risk patients.
